# IL-17A is increased in the serum and in spinal cord CD8 and mast cells of ALS patients

**DOI:** 10.1186/1742-2094-7-76

**Published:** 2010-11-09

**Authors:** Milan Fiala, Madhuri Chattopadhay, Antonio La Cava, Eric Tse, Guanghao Liu, Elaine Lourenco, Ascia Eskin, Philip T Liu, Larry Magpantay, Stephen Tse, Michelle Mahanian, Rachel Weitzman, Jason Tong, Caroline Nguyen, Tiffany Cho, Patrick Koo, James Sayre, Otoniel Martinez-Maza, Mark J Rosenthal, Martina Wiedau-Pazos

**Affiliations:** 1 Department of Medicine, David Geffen School of Medicine at UCLA and VA Greater Los Angeles Healthcare System, 650 Charles E. Young Dr. South, Los Angeles, CA 90095-1735, USA; 2 Department of Chemistry and Biochemistry, UCLA, 10833 Le Conte Ave, Los Angeles, 90024, USA; 3 Department of Medicine, David Geffen School of Medicine at UCLA, 10833 Le Conte Ave, Los Angeles, 90024, USA; 4 Department of Human Genetics, David Geffen School of Medicine at UCLA, 10833 Le Conte Ave, Los Angeles, 90024, USA; 5 Department of Microbiology, Immunology and Molecular Genetics, David Geffen School of Medicine at UCLA, 10833 Le Conte Ave, Los Angeles, 90024, USA; 6 Departments of Obstetrics & Gynecology and Microbiology, Immunology & Molecular Genetics, David Geffen School of Medicine at UCLA, 10833 Le Conte Ave, Los Angeles, 90024, USA; 7 Department of Biostatistics, UCLA School of Public Health, 10833 Le Conte Ave, Los Angeles, 90024, USA; 8 Department of Neurology, David Geffen School of Medicine at UCLA, 10833 Le Conte Ave, Los Angeles, 90024, USA

## Abstract

The contribution of inflammation to neurodegenerative diseases is increasingly recognized, but the role of inflammation in sporadic amyotrophic lateral sclerosis (sALS) is not well understood and no animal model is available. We used enzyme-linked immunosorbent assays (ELISAs) to measure the cytokine interleukin-17A (IL-17A) in the serum of ALS patients (n = 32; 28 sporadic ALS (sALS) and 4 familial ALS (fALS)) and control subjects (n = 14; 10 healthy subjects and 4 with autoimmune disorders). IL-17A serum concentrations were 5767 ± 2700 pg/ml (mean ± SEM) in sALS patients and 937 ± 927 pg/ml in fALS patients in comparison to 7 ± 2 pg/ml in control subjects without autoimmune disorders (p = 0.008 ALS patients vs. control subjects by Mann-Whitney test). Sixty-four percent of patients and no control subjects had IL-17A serum concentrations > 50 pg/ml (p = 0.003 ALS patients vs. healthy subjects by Fisher's exact test). The spinal cords of sALS (n = 8), but not control subjects (n = 4), were infiltrated by interleukin-1β- (IL-1β-), and tumor necrosis factor-α-positive macrophages (co-localizing with neurons), IL-17A-positive CD8 cells, and IL-17A-positive mast cells. Mononuclear cells treated with aggregated forms of wild type superoxide dismutase-1 (SOD-1) showed induction of the cytokines IL-1β, interleukin-6 (IL-6), and interleukin-23 (IL-23) that may be responsible for induction of IL-17A. In a microarray analysis of 28,869 genes, stimulation of peripheral blood mononuclear cells by mutant superoxide dismutase-1 induced four-fold higher transcripts of interleukin-1α (IL-1α), IL-6, CCL20, matrix metallopeptidase 1, and tissue factor pathway inhibitor 2 in mononuclear cells of patients as compared to controls, whereas the anti-inflammatory cytokine interleukin-10 (IL-10) was increased in mononuclear cells of control subjects. Aggregated wild type SOD-1 in sALS neurons could induce in mononuclear cells the cytokines inducing chronic inflammation in sALS spinal cord, in particular IL-6 and IL-17A, damaging neurons. Immune modulation of chronic inflammation may be a new approach to sALS.

## Background

Amyotrophic lateral sclerosis (ALS) is a paralyzing neurodegenerative disease, characterized by the loss of upper and lower motor neurons. A majority of cases are sporadic (sALS) and their cause remains unknown. Less than 10% of ALS cases are familial (fALS) with 20% of these cases linked to various mutations in the Cu/Zn mutant superoxide dismutase 1 (SOD-1) gene [1]. SOD-1 is an ubiquitous small cytosolic metalloenzyme that catalyzes the conversion of superoxide anions to hydrogen peroxide [2]. A subset of familial ALS cases is characterized by mutant SOD-1 protein aggregates in neuronal inclusions [3], which have toxic properties and occur selectively in motor neurons. Recently, inclusions with misfolded SOD-1 forms [4] and a wild-type SOD-1 sharing aberrant conformation and pathogenic pathway with mutant SOD-1 [5] have also been identified in sporadic ALS spinal cord motor neurons, suggesting the possibility that misfolded SOD-1 auto antigens stimulate inflammation in sporadic ALS as well.

SOD-1 mutations have diverse effects on the structure, functional activity and native stability of SOD-1, but a common pathway has been proposed through the formation of SOD-1 aggregates in the spinal cords of patients expressing SOD-1 mutations [6]. Emerging evidence suggests that protein misfolding and aggregation might be a common pathophysiologic link between sALS and fALS. In symptomatic transgenic mice that over express mutant SOD-1, a number of misfolded forms of SOD-1 are present in the spinal cords including those that expose regions of SOD-1 normally buried such as the dimer interface, and some of these forms have been found in patients. An altered SOD-1 species was found within the anterior horns of sALS patients that likely originated from misfolded wild type SOD-1[7], and oxidation of wild type SOD-1 produced a misfolded protein with toxic properties of mutant SOD-1 [8]. Recently, abnormally folded SOD-1 has been detected in the spinal cord inclusions of a subset of sALS patients [4]. Structural studies of the inclusions found in the spinal cords of transgenic ALS mice show that they are largely composed of SOD-1 fibrils [9,10]. These forms likely occur due to a lack of bound metal cofactors, such as copper and/or zinc, and the normal inter subunit disulfide bond, the posttranslational modifications that are critical for the exceptionally high stability and solubility of SOD-1. Soluble SOD-1, upon removal of bound metals, can be rapidly converted to amyloid fibrils by the reduction of the intramolecular disulfide bond, even in a small fraction of the protein [11].

Increased serum and CSF concentrations of cytokines in neurodegenerative diseases, such as Huntington disease [12] and Parkinson disease [13], are considered important in the disease pathogenesis even before the disease onset. In addition, non-neuronal glial cells contribute to ALS disease mechanisms [14], which is supported by transgenic mouse studies. Inflammatory cytokines, prostaglandin E2 and leukotriene B4, inducible nitric oxide synthase and NO were found in astrocytes from the G93A-SOD-1 mouse, an important model of human fALS [15]. Furthermore, adult microglia from mutant SOD-1 transgenic mice released tumor necrosis factor-alpha [16], which may stimulate IL-6 production from astrocytes and microglia leading to reactive gliosis in pathophysiological processes in the CNS [17]. However, the role of cytokines is not well understood in sALS patients, although previous studies highlighted a number of abnormal chemokines and cytokines, including CCL2 (MCP-1), interleukin-6 (IL-6), tumor necrosis factor-α (TNF-α), and recently, interleukin-17 (IL-17) and interleukin-23 (IL-23) in patients [18]. As recently suggested [19], some of these immune factors such as IL-6, and interleukin-13 (IL-13) positive T-cells, and IL-17A described herein, may be also useful as blood biomarkers for ALS.

The active role played by the immune system in ALS is revealed by the disrupted blood-brain barrier and the presence of activated macrophages, microglia, dendritic cells, T cells and mast cells in the spinal cord of ALS patients [20,21]. Positron emission studies show microglial activation during all stages of the disease in the motor cortex and other areas of the brain correlating with disease severity [22]. Transgenic mice expressing mutant SOD-1 also show evidence of extensive microglial activation, such as the increased expression of pro-inflammatory factors including transforming growth factor-β1, TNF-α and macrophage-colony stimulating factor [23]. Therefore, the pathway by which misfolded or aggregated SOD-1 triggers an inflammatory response is crucial to the role of inflammation in ALS.

Here, we report the cytokines induced by wild type and mutant SOD-1 proteins in peripheral blood mononuclear cells of ALS patients and extend the relevance of these studies by examining the immunopathology in the spinal cord of confirmed ALS patients (deceased). To our knowledge, these studies are the first to investigate how aggregated WT SOD-1 can trigger IL-1β, IL-6 and IL-23 responses in human mononuclear cells, the cytokines participating in the induction of IL-17A. Our results suggest that the activation of chronic inflammation, including the IL-17A mediated pathway, a signature of autoimmune diseases such as multiple sclerosis [24], is also critical in ALS.

## Methods

### Study population

Blood specimens for cytokine testing were obtained under UCLA Institutional Review Board-approved protocols. Written informed consent was obtained from all patients or their representatives according to the Declaration of Helsinki. 32 ALS patients, 28 with sporadic ALS (mean age 57.8 years) and 4 familial ALS subjects, who do not have a known SOD-1 mutation (mean age 67 years), 10 normal controls (mean age 56.6 years), and 4 subjects with autoimmune disease (mean age 58.7 years) were enrolled in this study via the UCLA ALS clinic (Table 1). The patients were diagnosed as probable or definite ALS by the revised EL Escorial criteria [25] and electromyography. The mean score of the ALS functional rating scale of sALS patients was 32.3 ± 2.1 months and mean disease duration was 24.5 ± 3 months.

**Table 1:** Demographic and cytokine information

	sALS	fALS	Control	**Autoimmune disorder**^**a**^
n	28	4	10	4
Age (years)	57.8 (2.5)	67.0 (4.7)	56.6 (9.6)	58.7 (13.0)
M/F ratio	14/14	2/2	7/3	4/0
Duration (months)	24.5 (3.0)	16.0 (4.2)	N/A	N/A
FRS	32.3 (2.1)	31.5 (2.7)	N/A	N/A
IL-17A^b^	64.2%*	75.0%	0.0%*	100.0%
IL-17A^c^	5767.4 (2700.4)	937.3 (927.8)	7 (4.8)	9993.6 (1865.5)
IL-17A^d^	1145.4 (317.9)^+^	984 (581.6)	6.7 (2.1)^+^	2815 (861.7)
IL-17A^e^	1126.0 (320.1)^+^	1124.0 (594.9)	7.1 (2.4)^+^	2074.0 (709.8)

Data are expressed as mean (± standard error of mean)

a Patients with eczema or asthma

b IL-17% positive in the serum (sALS vs. healthy controls, p = 0.003)

c IL-17 (pg/mL) serum level (ALS patients vs. normal controls, p = 0.008)

d IL-17 (pg/mL) PBMC supernatant level (without stimulation)

e IL-17 (pg/mL) PBMC supernatant level (with stimulation by fibrillar WT SOD-1 (2 μg/mL))

* p < 0.003 sALS vs. control

^+ ^p < 0.01 sALS vs. control

### Spinal cord tissues and fluid

Immunohistochemistry was performed on paraffin-embedded thoracic spinal cord tissues of 8 sporadic ALS patients, 3 Alzheimer disease patients and 4 other subjects without neuropathology (Table 2), which were provided by the UCLA Brain Bank (four ALS, 3 Alzheimer disease, and 1 case without neuropathology) and the UCLA National Neurological AIDS Bank (NNAB) (four ALS cases and 3 cases without neuropathology). UCLA NNAB also provided frozen lumbar and thoracic spinal cord sections of 3 ALS patients and 3 control non-ALS subjects without neuropathology.

**Table 2:** Demographic and diagnostic data of deceased patient providing spinal cord tissues

Subject	Age (at death)	Sex	Pathological Diagnosis
**1**	75 years	female	Sporadic amyotrophic lateral sclerosis
**2**	83 years	male	Sporadic amyotrophic lateral sclerosis
**3**	83 years	male	Sporadic amyotrophic lateral sclerosis
**4**	50 years	male	Sporadic amyotrophic lateral sclerosis
**5**	66 years	male	Sporadic amyotrophic lateral sclerosis
**6**	64 years	female	Sporadic amyotrophic lateral sclerosis
**7**	64 years	female	Sporadic amyotrophic lateral sclerosis
**8**	54 years	male	Sporadic amyotrophic lateral sclerosis
**9**	67 years	male	Alzheimer disease, Braak stage VI
**10**	90 years	male	Braak stage VI
**11**	76 years	male	Braak stage I
**12**	37 years	male	Angiosarcoma of pleura, no metastases
**13**	78 years	male	No neuropathology
**14**	62 years	female	No neuropathology
**15**	46 years	Male	No neuropathology

### Immunohistochemistry and immunofluorescence

Paraffin-embedded sections were deparaffinized, peroxidase activity was blocked with 3% hydrogen peroxide in methanol for 10 min, and subjected to heat-induced antigen retrieval at 95°C for 25 min. After dual endogenous enzyme block, they were stained by primary and secondary antibodies using Dakocytomation Envision⊕ System. Frozen sections were fixed with 4% paraformaldehyde, permeabilized with 1% Triton X-100, blocked with 1% bovine serum albumin, incubated with primary antibodies overnight at 4°C and secondary antibodies for 1 h at 37°C [26]. Primary antibodies were rabbit or mouse anti-CD3, anti-CD4, anti-CD8, anti-CD68 (Dako), goat anti-IL-1β (Santa Cruz), goat anti-caspase 3 (Santa Cruz), mouse anti-IL-6 (Cymbus), mouse anti-IL-10 (DNAX), mouse anti-IL-17A (R&D Systems), goat anti-IL-17A (C20) (Santa Cruz), mouse anti-TNF-α (SantaCruz), mouse monoclonal anti-mast cell tryptase, clone AA1 (DakoCytomation), mouse anti-granzyme B, clone GrB-7(Chemicon), normal mouse or goat IgG. Secondary anti-mouse, anti-rabbit, or anti-goat antibodies were ALEXA-595 or ALEXA-488-conjugated (Invitrogen). Phalloidin was FITC or TRITC conjugated (Sigma). The preparations were independently examined by two observers (MF and GL) using the Olympus Research microscope with Hamamatsu camera or using Leica SP5X White Light Laser Confocal microscope.

### SOD-1 protein preparation and fibrillation

Wild-type and mutant SOD-1 (G37R, G93A, D101N) were expressed in *Saccharomyces cerevisae *and purified using a combination of ammonium sulfate precipitation and hydrophobic interaction, ion exchange and size exclusion chromatography on Sephadex G75 column [27,28,11].. Purified SOD1, also known as "As-isolated SOD1" (AI SOD-1), was demetallated to generate APO-SOD-1using multiple rounds of dialysis against EDTA. The metal content of APO- and AI- SOD1 was verified by Inductively-coupled plasma mass spectrometry (ICP-MS). Typically, AI SOD1 contained 2.5 equivalents of zinc and 0.5 equivalents of copper, while APO-SOD-1contained 0.5-0.8 equivalents of each metal per dimer. To convert apo-SOD-1 to fibrils, 50 μM protein was incubated in 10 mM potassium phosphate (pH 7) and 5 mM DTT in a total volume of 200 μL in a chamber of a 96-well plate, including a polytetrafluoroethylene (PTFE) ball (1/8 inch diameter) and 40 μM thioflavin T [11]. The cytokine and plate was constantly agitated in a Fluoroskan microplate instrument (Thermo) at 37°C and fibril formation was monitored by thioflavin T fluorescence using λ_ex _of 444 nm and λ_em _of 485 nm. Fibril formation was indicated by a sigmoidal growth in fluorescence and verified by electron microscopy. For co-incubation with PBMC's, the SOD1 fibrils were precipitated by centrifugation at 16,000*g *for 15 minutes and resuspended in 10 mM potassium phosphate at pH 7. In some cases, fibrils were sonicated for 10 minutes in a bath sonicator at room temperature. To exclude contamination with endotoxin, endotoxin concentrations were determined using the Limulus amebocyte lysate assay (LAL assay) (Associates of Cape Cod) by a quantitative kinetic assay. The endotoxin levels, 0.0448 ± 0.0000 pg/ml in the wild type superoxide dismutase 1, and 0.32 ± 0.08 pg/ml in the G37R superoxide dismutase 1, were below the concentrations active as a pyrogen.

### Cytokine assays in fluids

Peripheral blood mononuclear cells were separated from heparin-anaticoagulated blood by Ficoll-Hypaque gradient centrifugation. The supernatant media of overnight cultures stimulated using the indicated SOD-1 protein (2 μg/ml), amyloid-β 1-42 (2 μg/ml) or medium with DMSO (1:1,000) were tested by multiplexed bioassays for human cytokines, the High Sensitivity Human Cytokine Panel - Pre-mixed 13 Plex (Millipore). The Luminex-platform assay panel simultaneously quantified supernatant concentrations of human IL-1β, interleukin-2 (IL-2), interleukin-4 (IL-4), interleukin-5 (IL-5), IL-6, interleukin-7 (IL-7), interleukin-8 (IL-8), interleukin-10 (IL-10), interleukin-12 (IL-12), IL-13, interferon-γ, granulocyte-macrophage colony stimulating factor, TNF-α. Assay results, expressed in pg/ml, were obtained using a Bio-RAD BioPlex 200 dual laser, flow-based sorting and detection analyzer. IL-17A cytokine was assayed in peripheral blood mononuclear cell supernatant and serum by a human-specific ELISA (DuoSet) kit (R&D Systems). IL-23 was assayed by the Human IL-23 immunoassay (R&D Systems).

**Whole-genome expression analysis **was performed with the Affymetrix Gene Array ST 1.0. RNA from each sample was prepared using manufacturer recommended protocols and the Qiagen RNAEasy columns. Each sample was labeled using standard protocols and reagents from Affymetrix. Probes were fragmented and hybridized to the Affymetrix Human Gene 1.0 ST Array. Raw cel files generated from the Affymetrix Expression Console software were loaded into GeneSpring GX 10.0.2 software (Agilent) for analysis. We used the Robust Multi-array Analysis (RMA) probe summarization algorithm, with a transcript level of CORE.

**Statistical testing **was performed using the statistical software SPSS, Version 10.0, as follows: IL-17A in serum and IL-23 by non-parametric Mann-Whitney and Wilcoxon tests; supernatant cytokines by paired t-test analysis; the proportion of positive tests by the Fisher's exact test.

## Results

### Interleukin-17A in the blood of ALS patients

**Figure 1:**
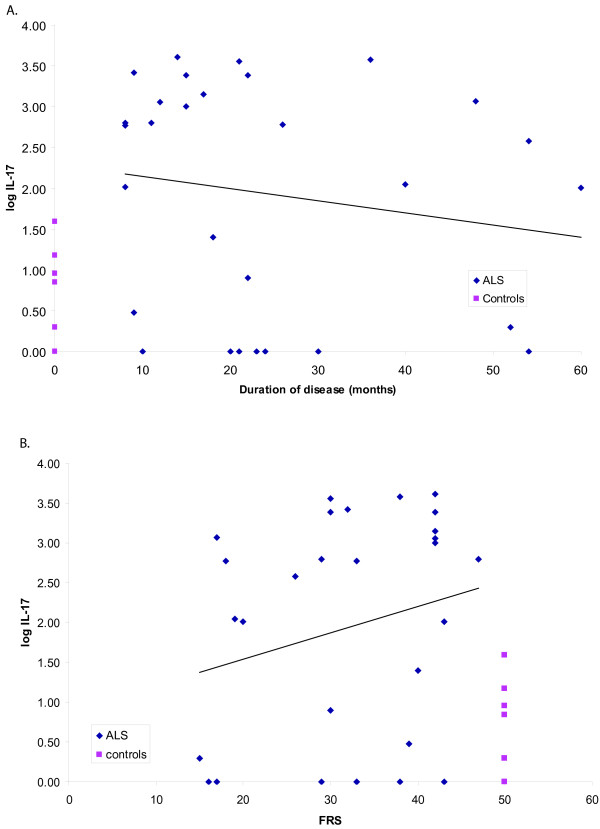
**Relation of blood IL-17A levels to disease duration and ALS functional rating scale (FRS)**. **(A) **Duration vs. log IL-17, correlation = -0.16, p = 0.38. **(B) **FRS vs. log IL-17, correlation = 0.23, p = 0.22.

The cytokine concentrations of IL-17A were assayed in 28 sporadic and 4 familial ALS patients, 10 normal control subjects, and 4 subjects suffering from episodes of eczema or asthma. Mean IL-17A serum concentrations of sALS patients (5767 ± 2700 pg/ml) were significantly higher (p = 0.008 by Mann-Whitney and Wilcoxon tests) than those in normal controls without autoimmune manifestations (7 ± 4.8 pg/ml) (Table 1). Mean IL-17A serum concentrations in fALS patients were 937 ± 927 pg/ml. The level of IL-17A was increased above the highest observed level in control subjects of 40 pg/ml in 64% of sALS patients, 75% fALS patients, 100% subjects with immune disorders and 0% normal controls (p = 0.003 sALS vs. healthy controls). The production of IL-17A in sALS PBMC's was constitutive, i.e., unlike other cytokines, it was not increased by SOD-1 stimulation over 18 hr: not stimulated 1145 ± 317.9 pg/ml; stimulated with fibrillar wild type SOD-1, 1,126 ± 320 pg/ml. In a cross-sectional analysis, IL-17 concentrations were highest in early patients and appeared to decrease with duration of illness and FRS (N.S.) (Figure 1A, B). However, the serum and PBMC supernatant concentrations of IL-17A in two patients followed over three months appeared to be fluctuating (data not shown).

### Immunopathology of the ALS spinal cord

**Figure 2:**
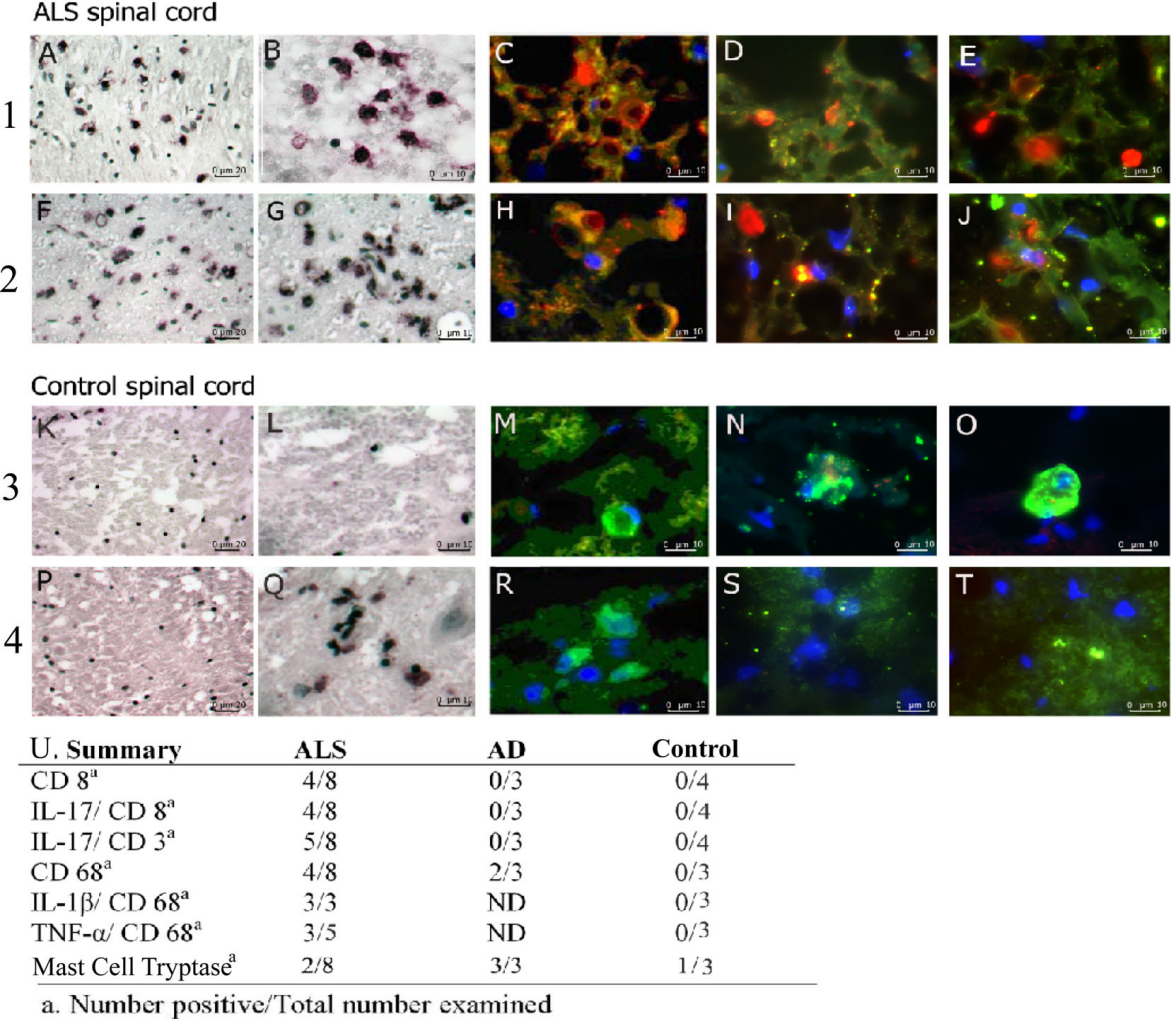
**IL-17A/CD8 T cells and TNF-α/CD68 cells infiltrate ALS spinal cord tissues**. Sections of thoracic spinal cord of five ALS patients (**A to J**) and 5 control subjects (two Alzheimer disease patients (**K, L, P, Q**) and 3 patients without neurological disease (**M, N, O, R, S, T**) were stained by CD8 antibody (**A to E)**, CD3 antibody **(K to O**) and CD68 antibody (**F to J; P to T**) using immunohistochemistry (IHC) (**A, B, F, G, K, L, P, Q**) or immunofluorescence (IFA) (**C, D, E, H, I, J, M, N, O, R, S, T**). The significant results were: **1**. CD8 in ALS gray matter: focal aggregates of IL-17A/CD8 T cells (red/brown by IHC **(A, B**) and red/green by IFA (**C, D, E**)) in 4/8 ALS spinal cords; **2**. CD68 in ALS gray and white matter: diffuse infiltration by TNF-α/CD68 cells (red/brown by IHC (**F, G)**, and red/green by IFA (**H, I, J**)) in 4/8 ALS spinal cords; **3**. CD3 in control gray matter: Lack of IL-17A on CD3 cells (no red staining by IHC **(K, L**) and IFA (**M, N, O**)) in 3 AD and 4 control spinal cords; **4**. CD68 in control gray and white matter: No TNF-α on CD68 cells (no red staining by IHC (**P, Q)**, and IFA (**R, S, T**) in 3 control spinal cords; IL-17A/mast cell tryptase-positive cells (Fig. 4E) were found in three of 5 ALS and 3 of 3 AD spinal cords. Staining experiments with normal mouse or goat IgG and a secondary antibody were negative with all specimens.

T cells, macrophages/microglia and mast cells were the immune cells found in ALS and control spinal cords. The ALS spinal cord was infiltrated in patchy fashion by T cells and mast cells in the gray matter, and diffusely by macrophages/microglia in the gray and white matter (Figure 2).

**Figure 3:**
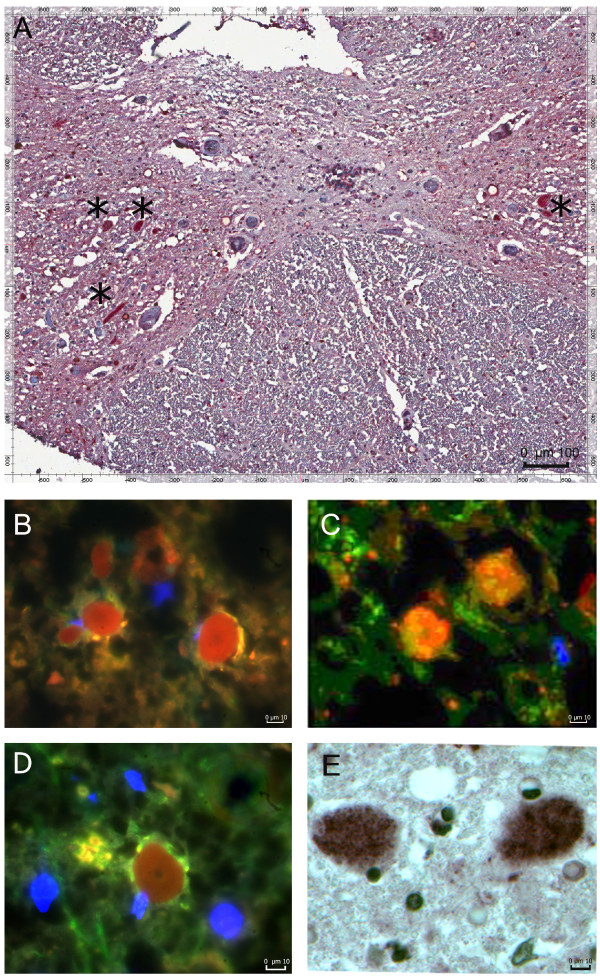
**IL-17A-positive cells are CD3 cells and mast cells localized in gray matter; CD3 T cells express granzyme B and caspase-3**. Immunohistochemistry **(A, E) **and immunofluorescence (**B, C, D)**. (**A**) IL-17A-positive cells (indicated by*) in the gray matter; (**B**) Granzyme B/CD3 (red/green); (**C**) caspase-3/CD3 (red/green); (**D**) IL-17A/CD3 (red/green); (**E**) IL-17A/mast cell tryptase-positive mast cells (red/brown).

In the gray matter, IL-17A was expressed in patchy fashion on several cell types: (1) CD8-positive CD3-positive T cells, (2) CD8-negative CD3-positive T cells, (3) mast cell tryptase-positive mast cells. Some CD3 cells expressed granzyme B or caspase-3 (Figure 3).

**Figure 4:**
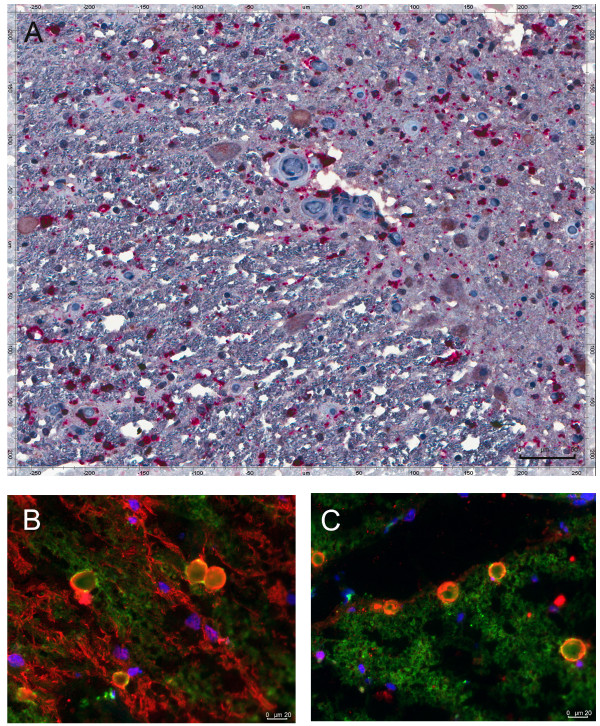
**IL-1β/CD68 macrophages infiltrate both the gray and the white matter of the ALS spinal cord**. Immunohistochemistry **(A)**; immunofluorescence **(B, C). (A) **IL-1β/CD68 (red/brown) macrophages in the gray and the white matter; **(B, C) **IL-1β/CD68 (red/green) macrophages.

**Figure 5:**
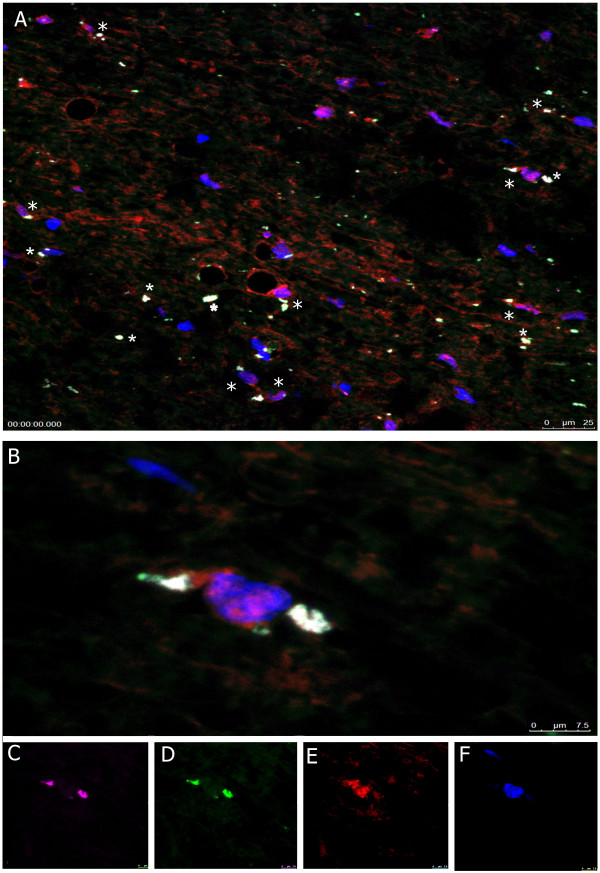
**TNF-α/CD68 macrophages overlie neurons**. Confocal microscopy of ALS spinal cord. **(A) **TNF-α/CD68 (magenta/green) macrophages overlie many NeuN/DAPI (red/blue) neurons and appear white (*) (40×); **(B) **Two macrophages (magenta/green) overlie a neuron (red/blue) (100×); **(C) to (F) **TNF-α (magenta); CD68 (green); neu-N (red); DAPI (blue).

The gray and the white matter were diffusely infiltrated by CD68-positive macrophages/microglia. These cells were IL-1α and TNF-α-positive (Figure 2 and 4), and many co-localized with neurons (Figure 5).

In summary: Two observers examined 87 slides stained by CD3, CD8, IL-17A, CD68, IL-1β, TNF-α and mast cell tryptase antibodies. IL-17A/CD3 T cells were found in five of 8 ALS spinal cords but not in any AD or control spinal cords; macrophages in 4 ALS spinal cords and two AD spinal cords; and mast cells in two ALS and three AD spinal cords (Figure 2U).

### Induction of IL-1β, IL-6 and IL-23 cytokines by fibrillar wild type and mutant SOD-1

**Figure 6:**
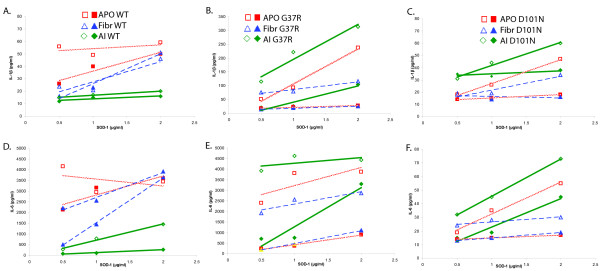
**Fibrillar and APO wild-type SOD-1, and APO and AI mutant forms of SOD-1 induce IL-1β and IL-6**. Peripheral blood mononuclear cells of two sALS patients were stimulated by the indicated SOD-1 proteins and cytokines were measured in the supernatant after 18 h. Note positive dose-response of IL-1β and IL-6 to fibrillar and APO wild-type SOD-1(**A, D**), and to APO and AI forms of G37R and D101N SOD-1 (**B, C, E, F**) (open symbols = patient 1; closed symbols = patient 2)

To clarify whether certain physical forms of SOD-1 could induce the cytokines IL-1β, IL-6 and IL-23, which can polarize T cells into the TH17 subset, we tested their induction by three forms of wild type and mutant SOD-1: as-isolated (AI), demetallated (APO), and fibrillar (Figure 6). These preparations had no significant levels of endotoxin (see Methods). IL-1β and IL-6 are the central pro-inflammatory cytokines and their induction by different physical forms of SOD-1 identifies these forms as responsible for inflammation. The dose-response experiment with PBMC's of two ALS patients and the wild type SOD-1 proteins showed induction by the fibrillar and APo forms, but not the AI form; and the experiment with the G37R and D101N SOD-1 mutant SOD-1 proteins, showed induction by the AI and APO forms, but not the fibrillar form.

IL-23 production was increased by stimulation with fibrillar SOD-1 of ALS patients' PBMC's (n = 6; mean ± S.E.M; not stimulated 52 ± 11.2 pg/ml, stimulated 123 ± 24.5 pg/ml; Wilcoxon p value = 0.0156) and control subjects' PBMC's (n = 3; mean ± S.E.M; not stimulated 27.6 ± 2.9 pg/ml, stimulated 188 ± 50 pg/ml; Wilcoxon p = .1) (data not shown).

**Figure 7:**
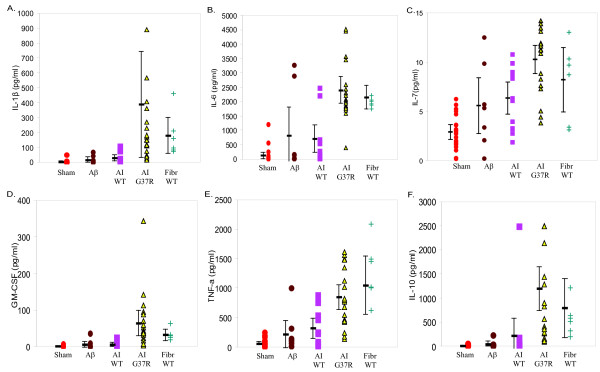
**Stimulation of the supernatant cytokines IL-1β, IL-6, IL-7, IL-10, GM-CSF and TNF-α by stimulation with sham, amyloid-β, AI WT, AI G37R and fibrillar SOD-1 in sALS patients**. Mutant SOD-1 induced 6 cytokines: IL-1β (P = 0.006; AI G37R vs. AI wild type SOD-1); interleukin-6 (P < 0.001; AI G37R vs. AI wild type SOD-1); IL-7 (P = 0.021; AI G37R vs. AI wild SOD-1); TNF-α (P < 0.001, AI G37R vs. AI wild type SOD-1); granulocyte-macrophage colony stimulating factor (P = 0.026, AI G37R vs. AI wild type SOD-1), and IL-10 (P = 0.026, AI G37R vs. AI wild type SOD-1).

To clarify the spectrum of cytokine responses in ALS patients, we tested induction of 13 supernatant cytokines in a sample of 29 patients by sham, amyloid-β, AI and fibrillar forms of wild type SOD-1, and the AI form of G37R SOD-1. The fibrillar wild type SOD-1 and AI G37R SOD-1, but not Aβ or AI wild type SOD-1, significantly increased six of the 13 tested cytokines in the supernatant media: IL-1β, IL-6, IL-7, TNF-α, granulocyte-macrophage colony stimulating factor, and IL-10 (Figure 7). Stimulation of control PBMC's with SOD-1 forms induced the same six cytokines (data not shown).

### Transcriptional stimulation by SOD-1 increases inflammatory cytokines in patients and the anti-inflammatory cytokine IL-10 in control subjects

**Figure 8:**
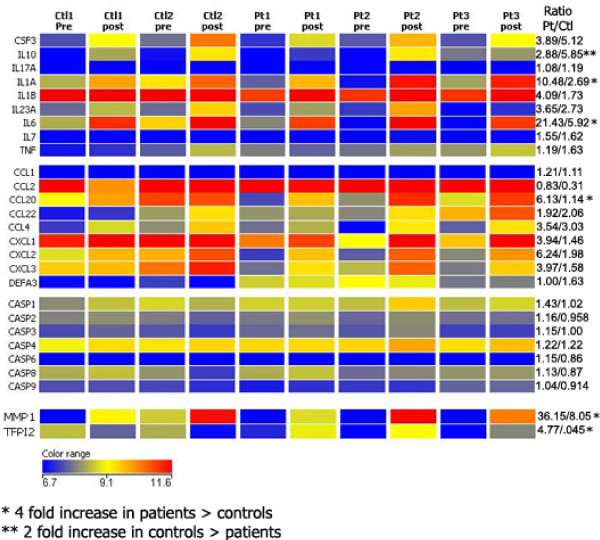
**Transcriptional upregulation by G37R SOD-1 of cytokines, chemokines, MMP1 and TFPI2**. The values after Robust Multi-array Analysis (RMA) are displayed for patients and control subjects and the ratio of values (patient/control) for each gene is shown. Note the cytokines (*) increased and those (**) decreased in patients in comparison to controls.

The responses to mutant SOD-1 were analyzed at the transcriptional level by microarray hybridization. The analysis of 28,869 transcript cluster identifications in peripheral blood mononuclear cells of 2 controls and 3 patients (stimulated 18 hr by mutant SOD-1) showed strong stimulation of 7 cytokines (IL-10, IL-23A, granulocyte macrophage colony stimulating factor, IL-1β, IL-1α, IL-6 and IL-7) in both patients and control subjects (Figure 8). Of these, the increase in transcription of IL-1α and IL-6 was ~ four-fold higher in patients compared to controls. In agreement with the constitutive production of IL-17A, its mRNA was not stimulated by G37R SOD-1. The chemokines CCL2, CXCL1, CXCL2, and CXCL3 were transcribed at a high level in patients as well as controls at baseline and after stimulation. G37R SOD-1 stimulated more CCL20 (5.5 fold), matrix metallopeptidase 1 (4.5fold), and tissue factor pathway inhibitor 2 (11.7 fold) in PBMC's of patients, as compared to control subjects. On the other hand, the anti-inflammatory cytokine IL-10 mRNA was stimulated more (2.0 fold) in PBMC's of control subjects, as compared to patients.

## Discussion

Our results show that, in a cross-sectional study, 64% of sALS and fALS subjects have strongly increased serum concentrations of the cytokine IL-17A, compared to normal subjects, and the concentrations of IL-17A fluctuate, which could result in false-negative results in some subjects. The spinal cord of deceased sALS patients show a milieu in which polarization of CD3 cells to IL-17A-producing cells can develop in response to products of macrophages, T cells and mast cells; including IL-1β, TNF-α, IL-6, IL-23, and probably eicosanoids (Figure 2 and 6). The cytokine IL-17A is pathogenic in inflammatory and autoimmune diseases such as multiple sclerosis [24], psoriasis, inflammatory bowel disease, systemic lupus erythematosus, and rheumatoid arthritis [29]. CD8 cells in gray matter might have a role in tissue destruction by cytotoxic cytokines, the cytotoxic molecules granzyme B, and nitric oxide (NO), resembling the role of CD8 cells in multiple sclerosis [24]. Although IL-17A is expressed on CD4 cells in the animal model of multiple sclerosis, experimental allergic encephalitis [24], IL-17A is expressed also on other cells, such as macrophages in asthma [30], CD8 cells in Behcet disease and psoriasis [31], and mast cells in rheumatoid arthritis synovium.

The ALS spinal cord is infiltrated by IL-17A-positive T cells and IL-17A-positive mast cells in gray matter and by TNF-α-positive macrophages/microglia in gray and white matter (Figure.2, 3, 4). Macrophages/microglia were found to co-localize with neurons, reminiscent of previously demonstrated large phagocytic cells surrounding atrophic neurons [32]. Although the identification of these cells as macrophages or microglia is not possible since both are CD68-positive, blood-derived macrophages may penetrate into the ALS spinal cord, as suggested by their presence around the vessels with disrupted ZO-1 junctions [32], and into Alzheimer disease brain, as shown by their invasion across brain endothelial cells with disrupted ZO-1 junction [33].

To clarify the induction of IL-17A in ALS patients, we focused attention on IL-1β, IL-6 [34], and IL-23, which are known to induce IL-17A and can be produced by macrophages and/or dendritic cells (Figure 6). The development of IL-17A-producing TH17 cells is initiated by transforming growth factor-β and IL-6, which induce phosphor-STAT-3 and the transcription factor RORγt, and is stabilized and expanded by the cytokines IL-21 and IL-23 [35,36]. Human TH17 cell differentiation requires IL-6, IL-1β and IL-21 or IL-23 [37]. In human studies, transforming growth factor-β has not been found to be essential [38]. In a recent mouse study, TH17 cells, which were induced by IL-1β, IL-6 and IL-23, were more pathogenic than those induced in presence of transforming growth factor-β [39]. The cytokines and chemokines required for TH17 polarization, IL-1α, IL-6, and CCL20, and matrix metalloproteinase 1, were transcriptionally stimulated more in ALS patients than in controls (Figure 8).

The presence of IL-17A in mast cells in the spinal cord of patients with ALS and Alzheimer disease (Figure 3) has not been previously reported. Mast cells together with macrophages produce eicosanoids [40], which are important in polarization of the TH17 subset [41]. Mast cells are emerging as master regulators with bi-functional role in both innate and adaptive immunity [42]. In the setting of autoimmunity, mast cells have a role in the initiation of the pathological immune response in experimental allergic encephalomyelitis through modulation of regulatory T cells into pathogenic Th17 cells [43]. Mast cells foster inflammation through the production of IL-6 and the shift of regulatory T cells to TH17 cells [44].

Fibrillar and APO forms of wild type SOD-1, but not the AI form, induced the key cytokines, IL-1β and IL-6, indicating their crucial role in inflammation of sALS patients (Figure 6). These autoantigens are likely present in the inclusions with non-native/misfolded forms of SOD-1, which are present in sporadic ALS spinal cords [4], and might be released from live or dying neurons [45,46] and be presented to autoimmune T cells by macrophages and dendritic cells.

Whole-genome expression analysis revealed that stimulation by SOD-1 increased in mononuclear cells of both patients and controls the transcription of cytokines, chemokines and matrix metallopeptidases. In patients' cells, however, the pro-inflammatory cytokines IL-1α and IL-6 were enhanced more and the anti-inflammatory cytokine IL-10 was enhanced less than in controls' cells (Figure 8). The chemokines expressed at a high level even before stimulation include CCL2 (MCP-1), CXCL1 (GROα), and CXCL3 (GROγ). The chemokine CCL20 (MIP-3α), a chemoattractant for CCR6, the marker of Th17 cells [47], was increased by SOD-1 stimulation more in patients' than in controls' cells. Matrix metallopeptidase 1, an effector of tissue remodeling [35], and tissue factor pathway inhibitor 2 were strongly stimulated in patients' cells, suggesting global pathology in ALS [48]. In agreement with the constitutive production of IL-17A in PBMC's, no increase in the transcription of IL-17A upon 18-hr SOD-1 stimulation was observed.

## Conclusions

**Figure 9:**
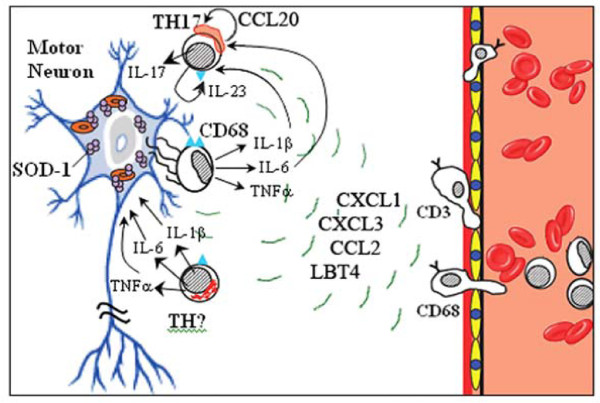
**Putative model of autoimmune inflammation in the ALS spinal cord**. Neurons, astrocytes and microglia induce chemokines when stimulated by aggregated superoxide dismutase 1. The chemokines (CXCL1, CXCL3 and CCL2) and leukotrienes attract monocytes to the neuropil. Aggregated SOD-1 stimulates macrophages and microglia to produce tumor necrosis factor-α, IL-1α, IL-1β, IL-6, and GM-CSF, which stimulate T cells to produce IL-23 and other cytokines. The cytokines IL-1α, IL-1β, IL-6, CCL20 and IL-23 polarize T cells and mast cells to produce IL-17A.

On the basis of these and previous observations [3,14,20], we propose the following immunopathogenesis of sporadic ALS (Figure 9): (a) aggregated wild type SOD-1 may induce chemokines and eicosanoids in a variety of neural cells, such as microglia, astrocytes and neurons; (b) The chemokines (CXCL1, CXCL3 and CCL2) and leukotrienes attract monocytes and T cells into the neuropil; (c) Aggregated SOD-1 stimulates macrophages and microglia to produce eicosanoids, tumor necrosis factor-α, IL-1β, and IL-6, which (d) stimulate T cells to produce IL-6, IL-23 and other cytokines and chemokines; (e) IL-1β, IL-6, CCL20 and IL-23 polarize CD8 (and CD4) T cells and mast cells to produce IL-17A; (f) CD8 T cells expressing granzyme B together with macrophages, mast cells, complement, NO, and other effector mechanisms cause neuronal apoptosis. Our results show a higher expression of IL-17A but lower expression of IL-10 in patients than controls, suggesting that the activation of regulatory T pathways is suppressed in patients, further suggesting a higher vulnerability of ALS patients to IL-17A-mediated damage. The discovery of IL-17A explains the chronic nature of inflammation in the ALS spinal cord and offers a new approach to therapy by immune modulation of inflammatory cytokines.

## Competing interests

The authors declare that they have no competing interests

## Authors' contributions

MF designed and supervised the study, analyzed the data, and wrote the paper. MC prepared SOD-1 proteins and helped to write the paper. AL, OM-M, MJR helped to write the paper. AE analyzed microarray data. ET, GL, EL, PTL, LM, ST, MMR, WJT, CN, TC, and PK performed the immune studies. JS performed statistical analysis. MWW examined the patients and helped to write the paper. All authors have read and gave approval for the publication of this final version of the manuscript.
